# Bilateral Knee Osteoarthritis Treated With Medial Open-Wedge High Tibial Osteotomy Using Two Types of β-Tricalcium Phosphate With Differing Placements in Each Knee: A Report of Two Cases

**DOI:** 10.7759/cureus.45427

**Published:** 2023-09-17

**Authors:** Hiromi Mochizuki, Tomokazu Yoshioka, Naoya Kikuchi, Masashi Yamazaki

**Affiliations:** 1 Department of Orthopaedic Surgery, Tsukuba Central Hospital, Ushiku, JPN; 2 Division of Regenerative Medicine for Musculoskeletal System, Institute of Medicine, University of Tsukuba, Tsukuba, JPN; 3 Department of Orthopaedic Surgery, Institute of Medicine, University of Tsukuba, Tsukuba, JPN

**Keywords:** bone remodeling, spherical porous β-tricalcium phosphate, unidirectional porous β-tricalcium phosphate, opening-wedge high tibial osteotomy, knee osteoarthritis

## Abstract

In medial open-wedge high tibial osteotomy (MOWHTO) for knee osteoarthritis, synthetic bone is commonly used as a replacement material for the opening gap. Unidirectional porous β-tricalcium phosphate (UDPTCP) and spherical porous β-tricalcium phosphate (SPTCP) have been widely used in this regard. In general, the two prostheses are placed parallel to the osteotomy opening gap. In this report, we discuss two cases involving a 63-year-old woman and a 51-year-old man who underwent MOWHTO for bilateral knee osteoarthritis. Both patients had experienced bilateral knee pain. In both patients, UDPTCP was placed anteriorly and SPTCP was placed posteriorly in one knee, with the placement reversed in the other knee. The remodeling of each type of β-TCP was evaluated using CT immediately after the surgery and one year postoperatively. The postoperative corrective loss and clinical outcomes were also evaluated. Remodeling with β-TCP was found to be faster with UDPTCP than with SPTCP, even though the anteroposterior placement differed laterally in each patient. Furthermore, there was no correction loss, and the clinical outcomes were comparable, regardless of the placement of β-TCP.

## Introduction

A medial open-wedge high tibial osteotomy (MOWHTO) is an effective method for the surgical treatment of medial unicompartmental knee osteoarthritis or osteonecrosis of the knee, and it has been associated with good clinical outcomes [1]. In MOWHTO, an osteotomy of the proximal tibia is performed, and two synthetic bones are placed in the opening gap, one at the front and the other at the back. These resorbable bone substitutes have sufficient strength and internal fixation materials to enable early weight-bearing [2]. Synthetic bones include hydroxyapatite and β-tricalcium phosphate (β-TCP), among which spherical porous β-tricalcium phosphate (SPTCP) is widely used [3]. SPTCP with a porosity of 60% and interconnected pores (100-400 μm in diameter), which are not arranged in one direction, is an artificial bone with excellent stability and bone remodeling capacity and is resorbed and replaced with the bone after implantation [4]. Unidirectional porous β-tricalcium phosphate (UDPTCP) is a novel porous artificial bone with a porosity of 57 ± 5% and composed of communication holes 25-300 μm in diameter, which are arranged in one direction. UDPTCP produces an appropriate balance between bone formation and material resorption because of its unique frosted columnar structure [5]. UDPTCP has been linked with excellent clinical outcomes in orthopedic surgery [6-8]. Kikuchi et al. [9] reported that bone remodeling occurred earlier in cases with UDPTCP than in cases with SPTCP placed in the open gap for OWHTO.

In this report, we present two cases in which MOWHTO was performed for bilateral knee osteoarthritis, with different anteroposterior placements of two different types of β-TCP in each knee.

## Case presentation

Two patients who underwent MOWHTO were evaluated. Case 1 was a female who presented to the orthopedic outpatient clinic complaining of right knee pain. She had undergone MOWHTO on the right knee at the age of 63 years. After right knee surgery, left knee pain appeared and gradually worsened, necessitating a left knee MOWHTO at the age of 64 years. Case 2 was a male who presented to the orthopedic outpatient clinic complaining of bilateral knee pain. He had undergone MOWHTO on the left knee at the age of 51 years and on the right knee at the age of 53 years. Both patients’ demographics and their preoperative radiographic parameters, including femorotibial angle (FTA), medial proximal tibial angle (MPTA), posterior tibial slope (PTS), and opening gap, are presented in Table 1.

**Table 1:** Demographic and preoperative radiographic data of both cases (age shown refers to age at the time of each surgery) SPTCP: spherical porous β-tricalcium phosphate; UDPTCP: unidirectional porous β-tricalcium phosphate. U-S: UDPTCP was implanted anteriorly and SPTCP was implanted posteriorly. S-U: SPTCP was implanted anteriorly and UDPTCP was implanted posteriorly; FTA: femorotibial angle; MPTA: medial proximal tibial angle; PTS: posterior tibial slope

Case	Age, years	Sex	Body mass index, kg/m^2^		FTA (°)	MPTA (°)	PTS (°)	Opening gap (mm)
1	63	Female	32.1	U-S	177.3	89.7	13.6	10.0
	64			S-U	178.3	86.5	14.4	12.0
2	51	Male	26.7	U-S	178.0	87.7	9.7	13.0
	53			S-U	179.3	86.5	12.1	14.0

Both cases were operated on and evaluated as described below.

At the time of surgery, a 7-cm longitudinal incision was made on the proximal-medial side of the tibia, and an osteotomy was performed. A bone saw and chisel were used for biplane osteotomy: one plane from the proximal medial side of the tibia toward the fibular head, and the other plane parallel to the tibial shaft behind the tibial tuberosity. The gap was gradually opened until the planned preoperative opening distance was achieved. The target for the percentage of mechanical axis was approximately 62.5% [10]. UDPTCP (Affinos®, Kuraray Co., Tokyo, Japan) and SPTCP (Osferion 60®, Olympus Thermo Biomaterials, Tokyo, Japan) were used as spacers in the opening gap. Each section was cut into wedges and placed parallel to the anterior and posterior parts. The right or left knee was implanted with UDPTCP anteriorly and SPTCP posteriorly and was defined as the U-S knee. The other knee was defined as the S-U knee, with SPTCP implanted anteriorly and UDPTCP posteriorly. A locking compression plate (TriS Medial HTO Plate System, Olympus Thermo Biomaterials, Tokyo, Japan) was used for internal fixation. Both patients used a cane to help with weight-bearing for one week, followed by partial weight-bearing. Full weight-bearing was allowed four weeks postoperatively.

**Figure 1:**
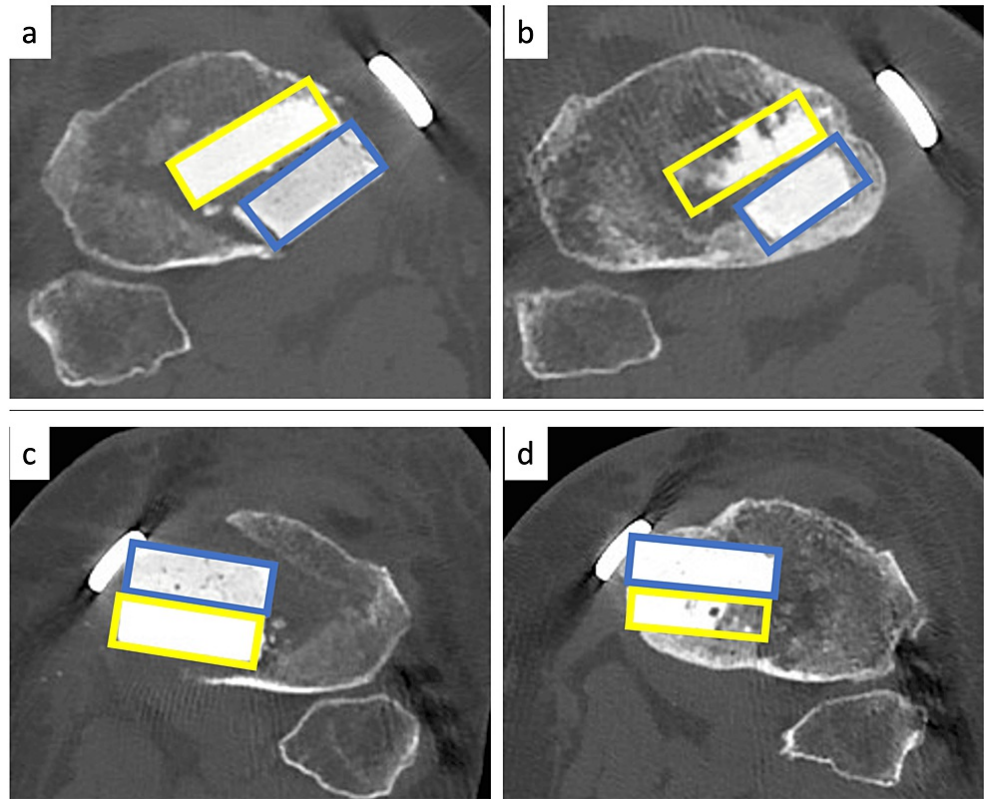
CT findings CT images of the center of the osteotomy plane for Case 1 show the mean CT value (HU) of the area implanted with UDPTCP (yellow rectangle) and the area implanted with SPTCP (blue rectangle). CT images of the U-S knee at one week (a) and last observation (b). CT images of the S-U knee at one week (c) and last observation (d) CT: computed tomography; HU: Hounsfield units; SPTCP: spherical porous β-tricalcium phosphate; UDPTCP: unidirectional porous β-tricalcium phosphate: U-S: UDPTCP was implanted anteriorly and SPTCP was implanted posteriorly; S-U: SPTCP was implanted anteriorly and UDPTCP was implanted posteriorly

To evaluate bone remodeling, CT was assessed one week and one year postoperatively. CT images parallel to the osteotomy plane were acquired using the method described by Tanaka et al. [11]. The image at the center of the osteotomy plane was divided into two regions, UDPTCP and SPTCP, and the CT values (Hounsfield units; HU) of each region were analyzed using Osirix® (Pixmeo Inc., Geneva, Switzerland) (Figure 1). To evaluate correction loss, the following radiographic parameters were measured: FTA, MPTA, and PTS at one week and one year postoperatively. Correction loss was defined as the numerical difference between the values of the radiographic parameters obtained one week and one year postoperatively (specifically, the value at one year postoperatively minus the value at one week postoperatively). Clinical outcomes were evaluated using the Japanese Orthopaedic Association (JOA) score for knee osteoarthritis [12] preoperatively and one year postoperatively.

**Figure 2:**
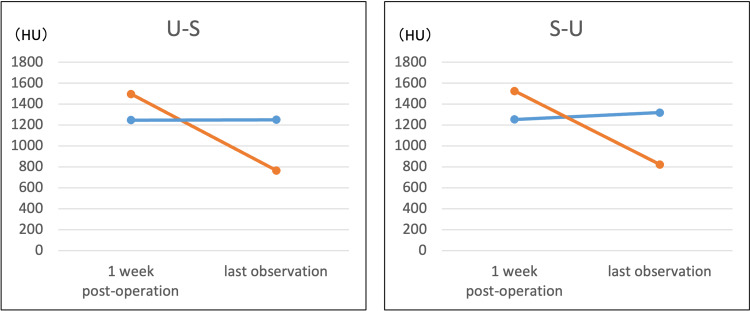
CT attenuation values (HU) of UDPTCP (red) and SPTCP (blue) in Case 2 at one week and last observation postoperatively CT: computed tomography; HU: Hounsfield units; SPTCP: spherical porous β-tricalcium phosphate; UDPTCP: unidirectional porous β-tricalcium phosphate: U-S: UDPTCP was implanted anteriorly and SPTCP was implanted posteriorly; S-U: SPTCP was implanted anteriorly and UDPTCP was implanted posteriorly

**Figure 3:**
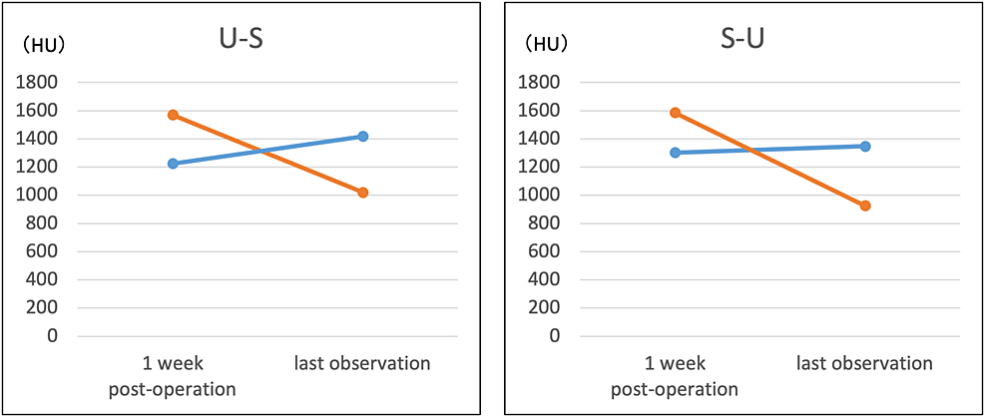
CT attenuation values (HU) of UDPTCP (red) and SPTCP (blue) in Case 1 at one week and last observation CT: computed tomography; HU: Hounsfield units; SPTCP: spherical porous β-tricalcium phosphate; UDPTCP: unidirectional porous β-tricalcium phosphate: U-S: UDPTCP was implanted anteriorly and SPTCP was implanted posteriorly; S-U: SPTCP was implanted anteriorly and UDPTCP was implanted posteriorly

Postoperative radiographic parameters and correction losses of all knees are shown in Tables 2-3. The CT values of the areas implanted with UDPTCP and SPTCP one week after surgery and at the last observation in all knees are shown in Figures 2-3. The JOA scores improved from 70 to 85 for the U-S knee and from 80 to 85 for the S-U knee in Case 1; they improved from 75 to 90 for the U-S knee and from 70 to 80 for the S-U knee in Case 2.

**Table 2:** Postoperative radiographic parameters in both cases SPTCP: spherical porous β-tricalcium phosphate; UDPTCP: unidirectional porous β-tricalcium phosphate; U-S: UDPTCP was implanted anteriorly and SPTCP was implanted posteriorly; S-U: SPTCP was implanted anteriorly and UDPTCP was implanted posteriorly; FTA: femorotibial angle; MPTA: medial proximal tibial angle; PTS: posterior tibial slope

Case		FTA (°)	MPTA (°)	MPTA (°)	PTS (°)	PTS (°)
		1-year post-surgery	1-week post-surgery	1-year post-surgery	1-week post-surgery	1-year post-surgery
1	U-S	170.8	93.5	92.7	14.6	14.4
	S-U	167.7	96.7	96.1	13.7	13.5
2	U-S	170.6	95.0	93.7	12.5	12.2
	S-U	174.7	94.9	94.2	11.2	10.6

**Table 3:** Changes in the medial proximal tibial angle (MPTA) and posterior tibial slope (PTS) SPTCP: spherical porous β-tricalcium phosphate; UDPTCP: unidirectional porous β-tricalcium phosphate. U-S: UDPTCP was implanted anteriorly and SPTCP was implanted posteriorly; S-U: SPTCP was implanted anteriorly and UDPTCP was implanted posteriorly

Case		MPTA (°)	PTS (°)
				1	U-S	0.8	0.2
S-U	0.6	0.2		
2	U-S	1.3	0.3
	S-U	0.6	0.6

## Discussion

In both our patients, MOWHTO was performed for bilateral varus knee osteoarthritis, and two types of prostheses, UDPTCP and SPTCP, were placed anteriorly and posteriorly. In all knees, the CT values of UDPTCP were higher than those of SPTCP in the first postoperative week in both patients; however, at the last observation, they were lower than those of SPTCP. The CT values (HU) quantify the amount of X-ray attenuation and are defined as -1000 HU for air and 0 HU for water. Furthermore, the CT values for the proximal tibia after MOWHTO range from 100 to 200 HU, with values close to this range indicating bone remodeling [11,13]. Furthermore, there was no correction loss in any knee, and the clinical outcomes improved.

A previous report [14] has compared MOWHTO implanted with UDPTCP anteriorly and SPTCP posteriorly with MOWHTO implanted with SPTCP anteriorly and UDPTCP posteriorly in unilateral knee osteoarthritis and observed early bone remodeling with UDPTCP, regardless of anterior or posterior placement. In this report, the CT values were lower with UDPTCP than with SPTCP, regardless of the patient or anterior/posterior placement. The process of bone remodeling in synthetic bone is influenced by several key factors, including its porosity [15]. The porosities of UDPTCP and SPTCP were 57% and 60%, respectively. Knabe et al. [16] reported that a higher porosity of SPTCP was more favorable for bone formation, and Tanaka et al. [17] measured the CT values in MOWHTO using SPTCP with different porosities (60% and 75%). They found that SPTCP with 60% porosity showed higher CT values than cancellous bone six years after surgery, whereas SPTCP with 75% porosity showed the same CT values as cancellous bone and was completely resorbed and converted to bone.

In the present study, UDPTCP exhibited lower CT values than SPTCP at the last observation, although the porosity of UDPTCP was almost the same as that of SPTCP. Animal experiments have shown that blood permeates more rapidly in UDPTCP than in SPTCP because of its structure [18], and the structure of UDPTCP may contribute to early bone remodeling. There was no correction loss, and the clinical score improved at one year postoperatively in all knees. The compressive strength of UDPTCP was greater than 14 MPa parallel to the direction of the pores, whereas that of SPTCP was 20 MPa.

In the present study, MOWHTO with different anteroposterior placements of the prosthesis in each knee of the same patient was evaluated. No correction loss was observed in any of the four knees at one year postoperatively. A meta-analysis by Han et al. [19] has shown no difference in the rate of correction loss in MOWHTO with or without synthetic bone, whereas Takeuchi et al. [20] reported that in MOWHTO using a tibial bone model, the stress on the plate and lateral cortical hinge was reduced in groups in which SPTCP (60% porosity, 20 MPa compressive strength) was implanted anteriorly and posteriorly in the opening gap, compared with groups in which no synthetic bone was implanted. Tanaka et al. [15] reported no correction loss at two years postoperatively in MOWHTO, with the implantation of SPTCP with 60% porosity and 20 MPa compressive strength into the medial cortical defect, where the contact stress was the greatest, and SPTCP with 75% porosity and 3 MPa compressive strength into the trabecular bone defect.

This study has a few limitations: we evaluated only two patients, and the follow-up duration was relatively short. More case reports involving longer-term follow-ups are needed to enhance the generalizability of findings. Furthermore, few studies have investigated the biomechanics of synthetic bone, especially UDPTCP in MOWHO, and further investigation is needed to determine how UDTCP contributes to stability, and the use of two UDPTCPs in the opening gap should be carefully examined to determine whether correction losses occur.

## Conclusions

In our report, two cases of MOWHTO with two different prosthesis placements for bilateral knee osteoarthritis showed earlier bone remodeling with UDPTCP than with SPTCP, regardless of the placement position. In addition, there was no correction loss one year postoperatively, and the clinical scores improved in all four knees.
